# Building public health leadership: design, implementation and outcomes of the WHO European autumn school on quality of care and patient safety

**DOI:** 10.3389/fpubh.2026.1825901

**Published:** 2026-04-10

**Authors:** Valter R. Fonseca, José Miguel Diniz, Gkini Eleftheria, Yuliya Shcherbina, Ana Carolina Baptista, Daniel Saldanha Resendes, Alessandro Berionni, Lenio Capsaskis, Nurshaim Tilenbaeva, Damir Ivankovic, Thanos Myloneros, Constantina Vasileiou, Ana Marques, Blerta Maliqi, Gundo Weiler, Paulo Sousa, Joao Breda

**Affiliations:** 1 WHO Athens Quality of Care and Patient Safety Office, WHO Regional Office for Europe, Athens, Greece; 2 Public Health Unit, Local Health Unit of São José, Lisbon, Portugal; 3 RISE-Health, MEDCIDS, Faculty of Medicine, University of Porto, Porto, Portugal; 4 Public Health Service, Local Health Unit of Almada/Seixal, Almada, Portugal; 5 World Federation of Public Health Associations (WFPHA), Geneva, Switzerland; 6 Faculty of Health Sciences, Libertas International University, Zagreb, Croatia; 7 World Health Organization, Geneva, Switzerland; 8 World Health Organization, Copenhagen, Denmark; 9 NOVA National School of Public Health, Comprehensive Health Research Center, CHRC, NOVA University Lisbon, Lisbon, Portugal; 10 WHO Collaborating Center for Education, Research and Evaluation of Safety and Quality of Care, Lisbon, Portugal

**Keywords:** leadership, patient safety, problem-based learning, public health, quality of care, training

## Abstract

**Objectives:**

To describe the pedagogical design and implementation of the WHO European Autumn School on Quality of Care and Patient Safety, characterize participant profiles, evaluate feedback and learning outcomes, and identify lessons for leadership capacity building for quality of care and patient safety.

**Methods:**

A descriptive analysis of the program's design was conducted across three annual editions. The 2023 edition described aims and foundations, while the 2024 and 2025 editions were evaluated using a standardized post-course survey, analyzed descriptively and thematically.

**Results:**

The curriculum evolved from a foundational pilot (2023) to a practice-oriented model (2025) building on competence- and problem-based learning. Participation grew from 14 participants (9 countries) to 35 (23 countries) and participants consistently reported high satisfaction with the immersive full week program. The clarity, accessibility and relevance of the course materials and a strong people-centered approach was highlighted by 91 and 94%, respectively, of the participants in the 2025 edition.

**Conclusion:**

The first three editions of the Autumn School were perceived as relevant for strengthening system- and policy-level leadership for quality of care and patient safety. Future editions should expand hands-on application and incorporate longitudinal follow-up to assess sustained impact.

## Introduction

Quality of care and patient safety – core pillars of universal health coverage – remain major challenges for modern health systems. It is estimated 1 in 10 patients is harmed in healthcare (1), with 40% of this harm occurring in primary and ambulatory settings (2) and at least 50% of it being avoidable (3). The magnitude of low-value care and avoidable harm highlights this quality gap.

Despite global policy commitments and guidance from the World Health Organization (WHO) Global Patient Safety Action Plan 2021–2030 (4), implementation remains uneven across the WHO European Region. Only a third of the countries report having national quality of care and patient safety strategies or action plans (5). Substantial variations across countries in key quality of care dimensions, health system functions and population health outcomes (5) indicate current approaches are insufficient to drive the change required.

A WHO transformational vision has been proposed (6) built on two pillars – meaningful patient outcomes and systems thinking – and supported by three enablers: empowered leadership, transparent data and innovative solutions. This vision aligns with the 12 Essential Public Health Functions (7). The core competencies of public health professionals encompass surveillance, health protection and promotion, and governance – including health service quality and equity. Therefore, the Essential Public Health Functions provide a foundation for quality of care and patient safety leadership, as they require systematic data collection, stakeholder coordination, and population-level interventions.

However, translating such a vision into practice requires leadership competencies at system and policy levels. Structured programmes that equip public health professionals and policymakers with the competencies necessary to drive quality improvement and patient safety initiatives are limited. A comprehensive assessment of public health leadership education in the European Region revealed that training on quality of care for health workers remain insufficient, employ heterogeneous approaches and lack systematic evaluation of their effectiveness (8–10).

In recognition of these gaps, the Autumn School on Quality of Care and Patient Safety (Autumn School) was launched in 2023 by the WHO Athens Quality of Care and Patient Safety Office in collaboration with the WHO Collaborating Centre for Education, Research and Evaluation of Safety and Quality in Healthcare at the NOVA National School of Public Health, Lisbon, Portugal.

### Objectives

This study aims to:

1. Describe the pedagogical design and implementation, including how these reflect WHO work on quality of care and patient safety and aligns with the WHO Global Competency and Outcomes Framework for Universal Health Coverage.
2. Analyse the participation and evaluate participants' feedback, across the two latest editions.
3. Identify lessons learned and implications for leadership capacity building for quality of care and patient safety across the WHO European Region.

## Methodology

### Course design and implementation

The Autumn School on Quality of Care and Patient Safety was designed as an immersive, week-long, in-person learning programme that brings together key health system decision-makers, including country delegates from Ministries of Health, patient association representatives (in the third edition), and regional experts and academics specializing in quality of care and patient safety.

The course was developed within the framework of the WHO European Programme of Work 2020–2025 (‘'United Action for Better Health”) (11) and global commitments to Universal Health Coverage (12) and the 2030 Agenda for Sustainable Development (13), which recognize quality of care and patient safety as core components of resilient, equitable and people-centered health systems. It then evolved to reflect the changing health systems landscape as well as the Second WHO European Programme of Work 2025–2030 (14) and align with the core principles laid out.

**Figure 1:**
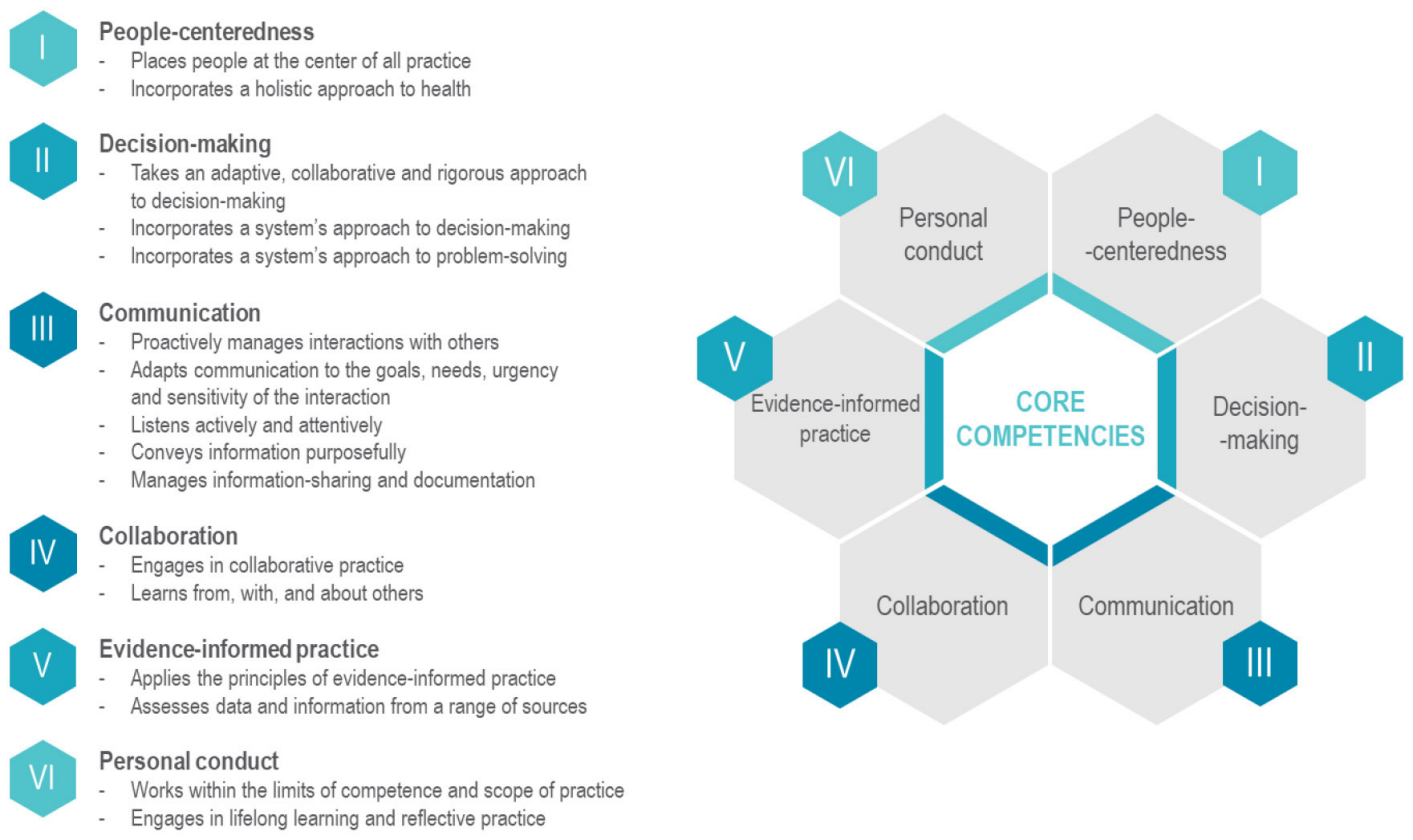
Competencies participants were expected to acquire throughout the course, by domain.

In the second and third editions of the Autumn School, the Quality of Care Policy Design Group Activity was the core pedagogical element, built around two complementary educational approaches: problem based learning (PBL) and competency based education (15) (CBE), both designed to foster engaged learning and skill development in a simulated health-care policy context (Figure 1).

PBL was used to promote critical thinking, problem-solving and self-directed learning skills (16) that are essential for effective health-care policymaking. Within the group activity, participants were placed in the role of active problem-solvers and asked to work on complex, real-world health-care challenges in quality of care and patient safety, based on publicly available country-specific health profiles (e.g., WHO Country Profiles on Quality of Care and Patient Safety, European Observatory for Health Systems and Policies, OECD). By requiring participants to analyze these challenges, apply evidence and develop contextually appropriate policy responses, the PBL design supported the development of tailored policy interventions that were both theoretically sound and practically viable and aligned with the complexities of health-care policymaking, where solutions must be both evidence-based and adequate to each setting. PBL was facilitated by WHO experts which guided group discussions and steered the selection of tools for quality of care policy formulation (such as, Plan-Do-Check-Act). CBE was integrated with PBL to shift the focus from knowledge acquisition to the demonstration of knowledge, skills and attitudes in real-world contexts (Figure 1).

### Course evaluation

We conducted a cross-sectional, mixed-methods descriptive study of the WHO European Autumn School on Quality of Care and Patient Safety across its first three editions. The inaugural 2023 edition was used as a foundational reference point to describe the program's aims, structure and core content, while the quantitative and qualitative analyses focused on the second and third editions (2024 and 2025).

Second and third editions featured a post-course survey, including both closed- and open-ended items. A survey was developed based on expert consensus and deployed through a web-based platform on the last day of the Autumn School. Participation in the survey was entirely voluntary and anonymous. Participants were informed by a disclaimer in the landing page of the survey that participation in the survey is regarded as tacit consent for their participation. As the survey did not impose any rule or behavior on the participants and was carried out in accordance with relevant guidelines and regulators for identity privacy and data protection, a medical ethics review of the study protocol was not pursued. Quantitative items were analyzed using descriptive statistics and, where appropriate, compared across years. Qualitative data from open-ended responses were analyzed by three independent researchers using thematic content analysis. The coding was compared and discussed until reaching consensus by the research team. Thematic analysis also helped to contextualize the quantitative findings.

## Results

### Design and conceptual evolution of the autumn school

Over its first three editions, the WHO Autumn School on Quality of Care and Patient Safety evolved through an iterative design-execution-review-improvement process, with each edition building on experience gained in earlier iterations, and considering the feedback provided from the organizing team, the participants and faculty (Table 1). While the overall aim of strengthening leadership for quality of care and patient safety remained unchanged, the program gradually developed its focus, structure coherence and depth in its practical orientation.

**Table 1:** Development across the three editions of the WHO autumn school.

Edition	Curriculum focus	Developments built from previous editions
1st Edition (Foundational) Lisbon, Portugal, 2023	Introduction of core foundations in quality of care and patient safety from a systems thinking and leadership perspective.	–
2nd Edition (Consolidation) < city>Lisbon < /city>, Portugal, 2024	Deeper integration of people-centeredness and policy-to-action orientation	Group work design through a problem-based learning and competence-based learning education was introduced.
3rd Edition (Advanced/Practice-oriented) Athens, Greece, 2025	Stronger focus on practical application, leadership for change, whole-system approach to quality of care and patient safety.	Multidisciplinary, inclusiveness and team-based work approaches, including patient representatives were incorporated.

The inaugural edition occurred in 2023 and served primarily to establish a common language and understanding around quality of care and patient safety, creating space for dialogue on key challenges and system-level priorities. It represented a formative step in developing a regional capacity-building initiative on quality of care and patient safety in the WHO European Region.

At this early stage, the Autumn School functioned as a pilot of a regional, in-person learning initiative. The focus was on exploring the feasibility and relevance of such a format, rather than on structured application or policy development. Opportunities for sustained peer exchange and hands-on work were limited, and the programme did not yet include a formal evaluation framework.

Experience and lessons from this first edition, along with informal participant feedback, informed the conceptual architecture and pedagogical approach of subsequent editions including a competency-based structure and the introduction of the problem-based learning (PBL) Policy Design Group Activity.

The second edition in 2024 reflected clear improvement from the first, with a more intentional approach to learning design. It was structured around five key themes, each forming the focus of one day of the programme: Foundations of Quality of Care; Governance and Measurement; Policy and Innovation; Safety and Resilience; and Patient-centeredness and Experience.

Another key development was the introduction of a structured, problem-based policy design group activity (Table 2). Working collaboratively on country-specific quality of care and patient safety issues throughout the week enabled participants to translate ideas into practice, while learning from one another's experiences.

**Table 2:** Outline of the policy development group activity during the course (2024 and 2025).

Day theme	Activities	Competences	
Day 1: Introduction	Background and objectives; methodology; group organization; materials; and expected outcomes. Experience-sharing and problem identification.	–	Domain III Domain IV Domain VI
Day 2: situation analysis	Facilitator wrap-up; identifying key healthcare challenges; accessing health system capabilities; analyzing stakeholders; gathering evidence; and identification of knowledge gaps. Production of a visual synthesis and a shared understanding of the root causes.	Domain I Domain V	
Day 3: policy development	Review of key challenges and objectives; brainstorming; policy conceptualization, including definition of actions targeting systemic barriers and integrate at least one enabler; policy drafting; policy refinement; and debrief and reflection.	Domain II Domain V	
Day 4: pitch crafting	Identifying key pitch elements; crafting the pitch; practice and refinement for clarity, brevity and impact; and wrap-up and next steps.	Domain I Domain II	
Day 5: policy dialogue	Presentation of policy policies; jury Q&A and feedback; debrief and reflection; and announcement of the winner.	–	

Each day combined conceptual input through keynotes and panels with skills-oriented workshops and structured PBL-based group work, allowing participants to apply tools and frameworks introduced during the course. The faculty was formed by international experts and academics, WHO experts and policy and decision-makers from different countries, combining both high-level technical expertise and real-life considerations, including enablers and barriers, from implementation experiences at the national level. Thus, participants were offered a clearer connection between conceptual discussions and practical challenges, supporting a more active and engaged learning environment.

Building on the foundations laid in earlier years, the 2025 edition's programme adopted a clearer transformational approach, the transformational vision for quality of care in the WHO European Region (6). The curriculum was organized along two pillars – outcomes that matter and systems perspectives – and three enabling dimensions: people, leadership and a drive for change; data and transparency; and digital solutions and innovation.

The agenda combined keynote lectures, panel discussions, roundtables and workshops mapped to these pillars and enablers with a PBL-based group activity in which participants developed quality of care and patient safety policy proposals.

Learning activities were more tightly aligned around this perspective, and its applied components were further refined to support deeper engagement with real-world challenges. Participants were better supported to connect strategic thinking with practical action, strengthening the balance between theory, experience and leadership for change (Table 2).

### Participation

Participants were officially nominated by their respective Ministries of Health and national authorities, ensuring the selection of professionals in key leadership and technical roles—such as quality coordinators, policy officers, senior clinicians, and health system managers—who are expected to apply the knowledge and tools acquired through the programme within their national quality of care and patient-safety initiatives.

**Figure 2:**
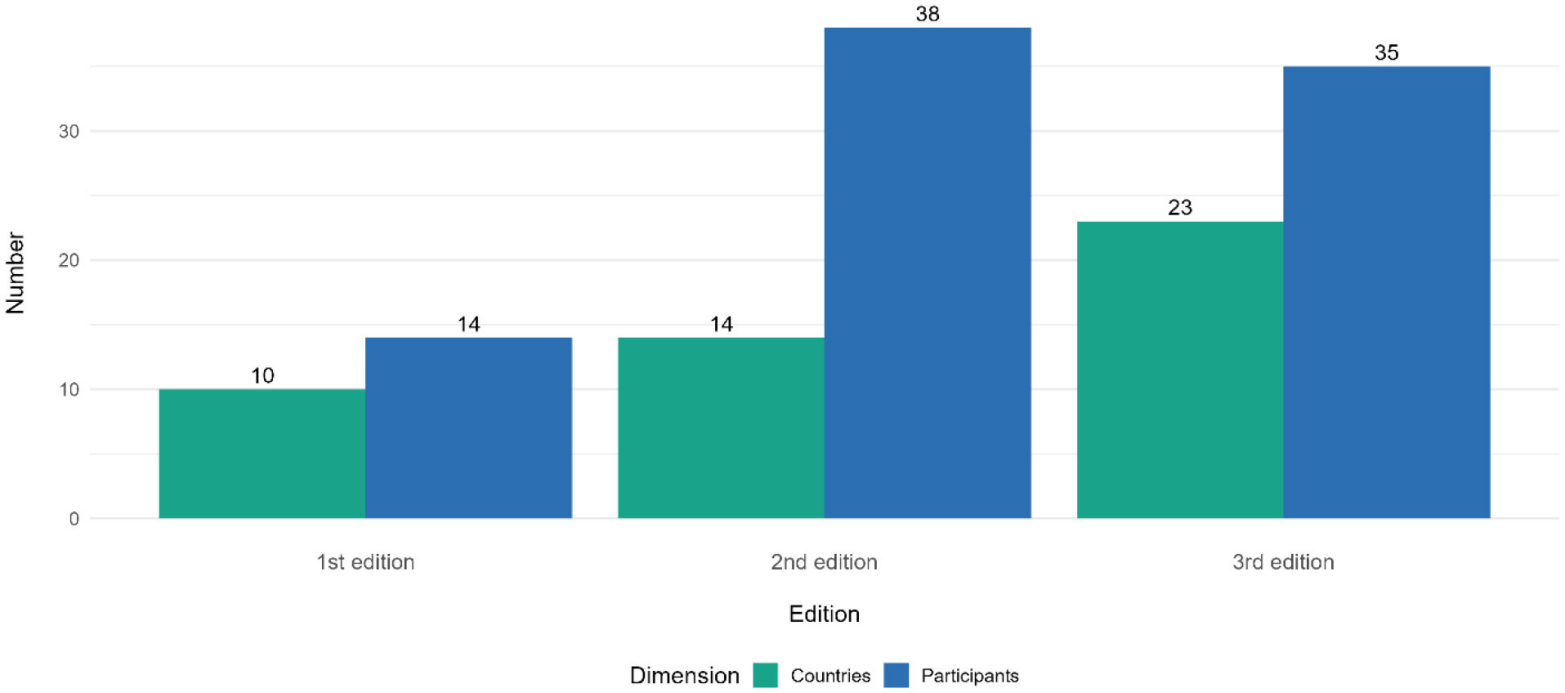
Number of participants and countries represented in the Autumn School, by edition. Countries represented in the 1st Edition (2023) – Albania, Bosnia and Herzegovina, Bulgaria, Cyprus, Malta, Portugal, Republic of Moldova, Republic of North Macedonia, Spain, Türkiye; Countries represented in the 2nd Edition (2024) – Albania, Bosnia and Herzegovina, Bulgaria, Cape Verde, Greece, Hungary, Malta, Montenegro, Portugal, Republic of Moldova, Republic of North Macedonia, Romania, Spain, Türkiye; Countries represented in the 3rd Edition (2025) – Albania, Armenia, Bosnia and Herzegovina, Bulgaria, Croatia, Cyprus, Czechia, Estonia, Georgia, Greece, Hungary, Kazakhstan, Kyrgyzstan, Latvia, Lithuania, Malta, Montenegro, Republic of Moldova, Republic of North Macedonia, Romania, Spain, Tajikistan, Türkiye.

The number of countries delegating representatives increased from 10 in the first edition to 23 in the third edition (Figure 2). In the second edition, from the 36 participants (94.7%) from which institutional affiliation was available the majority of respondents were from the public sector (*n* = 29, 80.6%), followed by international organizations (*n* = 4, 11.1%), and science and academia (*n* = 2, 5.6%), and others (*n* = 1, 2.8%). In the third edition, from the 33 participants (94.3%) data on which institutional affiliation was available, the majority of respondents were from the public sector (*n* = 27, 81.8%), followed by international organizations (*n* = 2, 6.1%) and one participant for each of the following categories: patient representative, science and academia, and others.

### Participants' feedback

#### 2024 Edition

**Figure 3:**
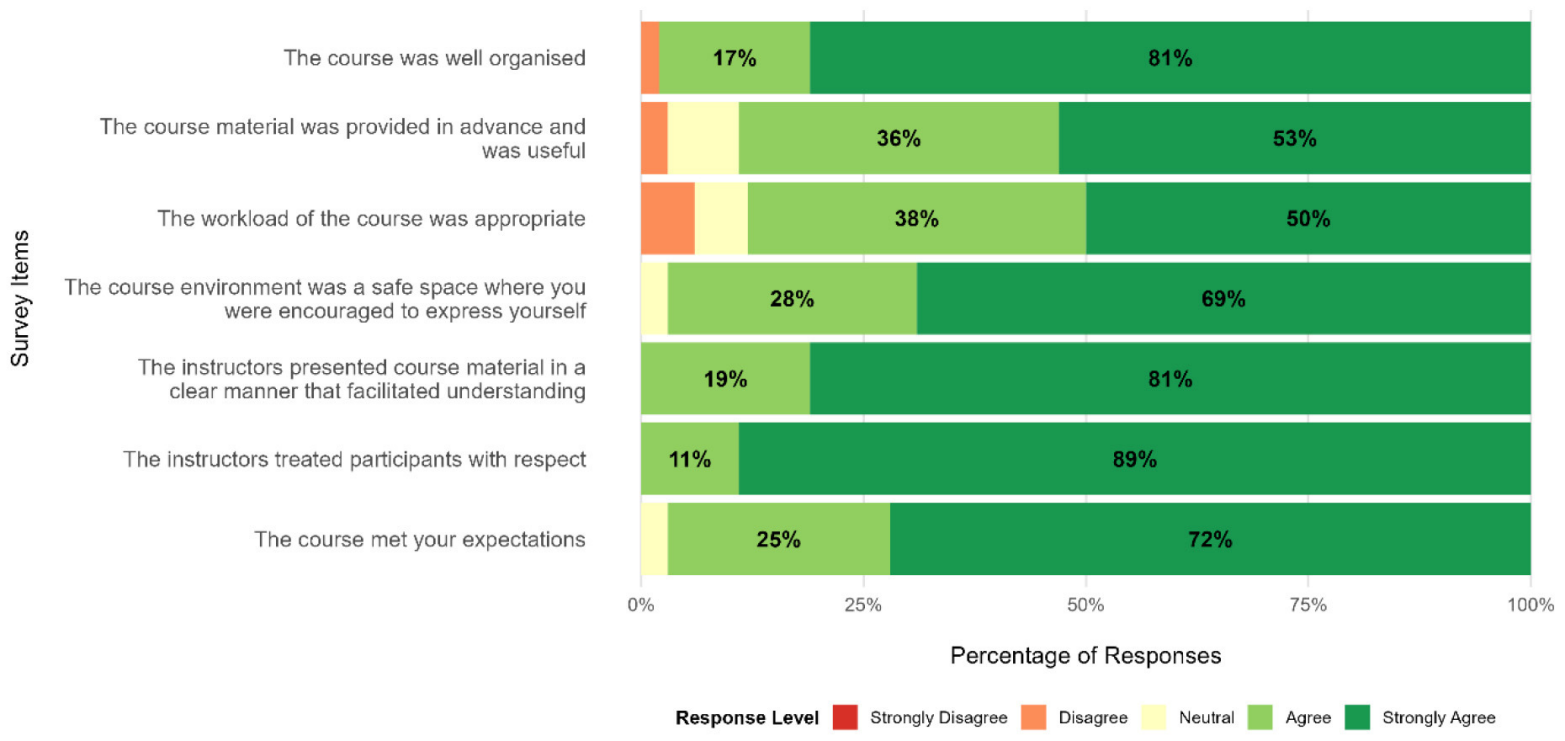
Responses of participants of the 2024 edition on topics of general satisfaction, by survey item. For readability purposes, options with less than 10% of the answers were omitted. Results on “Knowledge Acquisition” and “Knowledge Application” are provided in Supplementary Figures 1, 2.

From a total of 38 participants, 36 (95%) responded to the survey. Survey asked about general satisfaction, knowledge acquisition and knowledge application. Figure 3 presents the results of the end of course survey regarding “General Satisfaction”.

Participants reported high levels of satisfaction with the course where a large majority indicated agreement (responses of “Agree” or “Strongly Agree”) with statements related to course being well organized (35/36; 98%), timeliness and adequacy of the provided materials (32/36; 89%), and appropriateness of the course workload (32/36; 88%). Furthermore, 97% (35/36) of participants agreed that the course environment constituted a safe space that encouraged open expression. All 36 respondents agreed that instructors presented the course material clearly and treated participants with respect. Finally, 97% (35/36) of participants reported that the course met their expectations.

#### 2025 Edition

**Figure 4:**
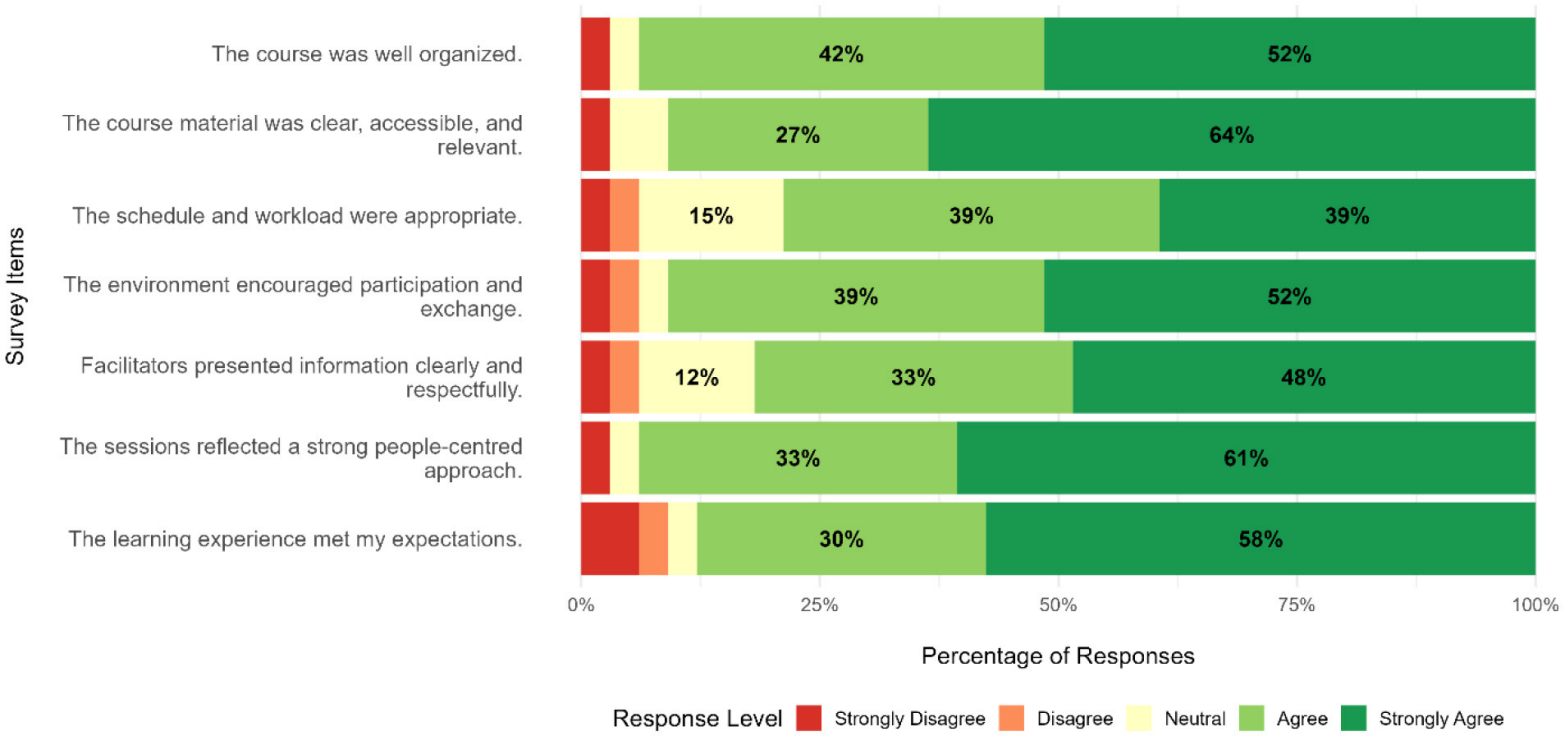
Responses of participants of the 2025 edition on topics of general satisfaction, by survey item. For readability purposes, options with less than 10% of the answers were omitted. Results on “Domain I: Outcomes that Matter”, “Domain II: Systems Perspective”, “Domain III: People, Leadership, and a Drive for Change”, “Domain IV: Data and Transparency”, “Domain V: Digital Solutions and Innovation” and “Domain VI: Collaboration” are provided in Supplementary Figures 3–8.

From a total of 35 participants, 33 (94%) responded to the survey. Figure 4 presents the results of the end of course survey regarding “Satisfaction and Knowledge Gains”.

Participants also reported high levels of satisfaction with the course. Most participants expressed agreement (responses of “Agree” or “Strongly Agree”) with statements regarding course being well organized (31/33; 94%), the clarity, accessibility and relevance of the course material (30/33; 91%) and the adequacy of the schedule and workload (26/33; 78%). Moreover, 91% (30/33) agreed the environment encouraged participation and exchange, while 81% (27/33) concurred (“Agree” or “Strongly Agree”) the facilitators presented information clearly and respectfully. Importantly, 94% (31/33) of the responders agreed the sessions reflected a strong people-centered approach. Lastly, 88% (29/33) of participants stated the course met their expectations.

Participants in the 2025 edition provided open-ended reflections on their key takeaways allowing the identification of lessons learned from these initiatives for forthcoming editions. Thematic analysis identified a strong emphasis on compassion and meaningful engagement with patients and families, underscoring that quality improvement is effective only when it reflects outcomes that matter to people. Participants reiterated the importance of reliable measurement and thoughtful interpretation of data to ensure that targets translate into real impact. Emerging challenges, such as workforce shortages, demographic change, climate pressures and the rapid evolution of digital technologies and AI, were noted as areas requiring sustained attention and evidence-informed action. Across all contributions, leadership, collaborative culture and continuous learning were identified as essential for strengthening health systems and supporting improvement at local and national levels.

## Discussion

This study describes the design, evolution and evaluation of the first three editions of the WHO European Autumn School on Quality of Care and Patient Safety, a capacity-building initiative aimed at strengthening system- and policy-level leadership for quality of care and patient safety. Across its initial iterations, the program evolved from a foundational pilot into a structured, competency-based and practice-oriented model explicitly aligned with the WHO transformational vision for quality of care and the Global Competency and Outcomes Framework for Universal Health Coverage.

This paper on the Autumn School contributes to a relatively underdeveloped area of the literature. While competency-based curricula in quality improvement and patient safety have been widely implemented in clinical education settings, most published initiatives focus on direct care provision rather than system stewardship and policy leadership. In contrast, the Autumn School was intentionally designed around competencies required for governance, intersectoral coordination and strategic decision-making. By integrating competency-based education with problem-based learning in a simulated policy environment, the program addresses a critical gap between conceptual frameworks for quality of care and their operationalization at system level (17). Evidence from comparable capacity-building initiatives suggests that structured training can lead to measurable improvements in knowledge. For example, a proof-of-concept patient safety training program implemented in North Macedonia showed significant gains in knowledge test scores immediately after the intervention (18). These findings suggest that well-structured, competency-oriented programs can support knowledge acquisition, while also highlighting the need for more robust evaluation designs to assess how learning is applied and sustained over time.

The progression observed across editions reflects an adaptive learning process consistent with principles of iterative program design. The shift from conceptual orientation (2023) to structured applied policy development (2024) and subsequently to a clearer alignment with a transformational systems perspective (2025) suggests increasing curricular coherence and maturity.

Importantly, participant feedback indicates that the applied components - particularly the policy design group activity - were central to perceived value. This aligns with broader evidence that sustainable improvements in quality of care and patient safety require leadership capable of operating across institutional boundaries, engaging multiple stakeholders and addressing structural determinants of health system performance (19). This highlights the strategic role of public health professionals as uniquely equipped to contribute to quality of care and patient safety. From foundational training on surveillance, data and monitoring, as well as governance, planning, financing, emergencies and community engagement, public health professionals are already equipped to drive quality improvement initiatives. As such, the Autumn School does not ask public health professionals to acquire entirely new capacities but challenges them to hone their skills and to take the lead in a topic traditionally governed by clinical actors.

Survey findings across the second and third editions demonstrated consistently high levels of satisfaction with course organization, learning environment and relevance. Although minor variation in specific indicators was observed between 2024 and 2025 (e.g., workload and facilitation ratings), overall agreement levels remained high. Such variation may reflect differences in cohort composition, increasing expectations as the program matured, and the more demanding applied format of later editions.

The thematic analysis of open-ended feedback reinforces the program's alignment with contemporary quality-of-care discourse. Participants emphasized meaningful patient outcomes, reliable measurement, system thinking and collaborative leadership—core elements of the WHO transformational vision (6) and of high-quality health systems literature. The recurrence of these themes suggests that the curriculum successfully foregrounded system-level perspectives rather than isolated quality improvement tools. Notably, emerging system pressures—such as workforce constraints, demographic shifts and digital transformation—were also identified by participants, indicating that discussions extended beyond technical quality frameworks toward broader health system resilience.

At the same time, the evaluation presented in this paper has important limitations. Outcomes were assessed primarily through self-reported perceptions and post-course assessments without baseline measurement or longitudinal follow-up. As such, findings reflect perceived knowledge acquisition and relevance rather than objectively demonstrated or sustained competency development. This limitation is common in short-term leadership training evaluations but underscores the need for more robust impact assessment approaches. Future evaluations should consider pre–post designs, longitudinal follow-up and exploration of downstream effects on policy development or governance practices.

Implications for future program development emerge from this analysis and from existing literature on leadership and adult learning. First, continued emphasis on applied, experiential components are warranted, as adult learning theory consistently demonstrates that practical engagement strengthens retention and transfer of competencies. Further refinement of collaborative learning components—particularly problem-based learning and policy design activities—could strengthen participants' ability to apply concepts in complex, real-world settings, while addressing the additional challenges of health systems identified by the thematic analysis (e.g., workforce constraints and demographic shifts). Second, structured peer exchange and cross-country dialogue should remain central, as transnational learning networks have been shown to support sustained policy diffusion and collaborative innovation. Future evaluations should focus on assessing longer-term impact, including how participants apply competencies within their organizations and whether participation contributes to observable changes in quality of care governance or policy development.

Follow-up activities with alumni, including periodic surveys or qualitative feedback, as well as co-design of future editions could provide a clearer picture of how learning translates into sustained change in practice and match participants' needs. Third, establishing a formal alumni network may facilitate longer-term exchange and provide a platform for monitoring how competencies are applied in national contexts.

The first three editions of the WHO European Autumn School demonstrated that a regionally anchored, competency-based and problem-based learning initiative can provide a structured platform for strengthening public health leadership in quality of care and patient safety. While further evaluation is required to assess sustained impact and behavior change, the program represents a promising model for linking global quality frameworks with practical system-level leadership development.

## Data Availability

The original contributions presented in the study are included in the article/Supplementary material, further inquiries can be directed to the corresponding author.
