# Supplementary material for: Building public health leadership: design, implementation and outcomes of the WHO European autumn school on quality of care and patient safety
Source: Front Public Health. 2026 Apr 10;14:1825901. doi: 10.3389/fpubh.2026.1825901 (PMC13106341; doi:10.3389/fpubh.2026.1825901)
Supplement: Supplementary file 1 [file Data_Sheet_1.docx]

Supplementary Data

***Supplementary Figure 1.*** *Responses of participants of the 2024 edition on knowledge acquisition across the six Autumn School domains.*


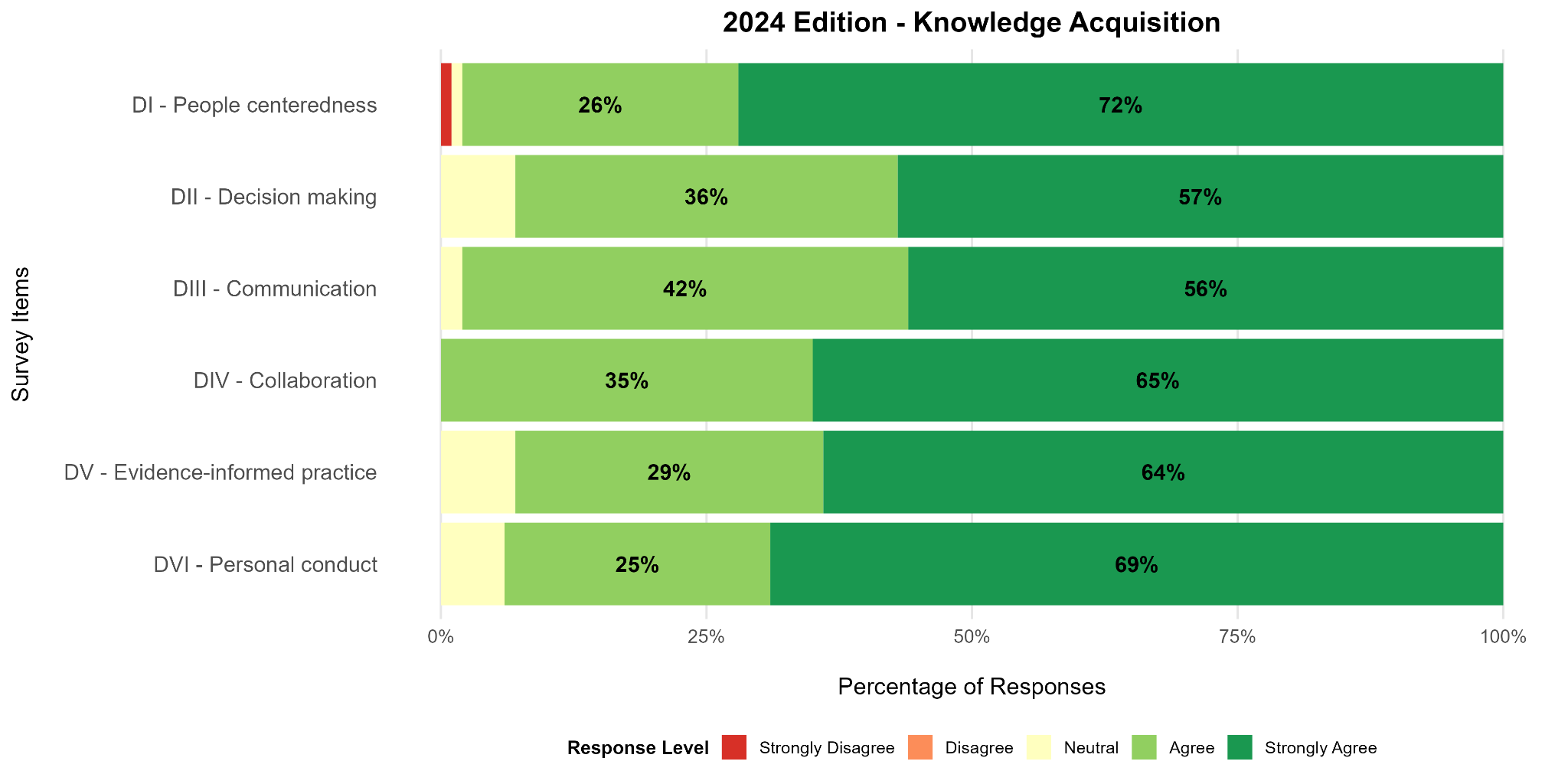


*For readability purposes, options with less than 10% of the answers were omitted.*

***Supplementary Figure 2.*** *Responses of participants of the 2024 edition on knowledge application across the six Autumn School domains.*


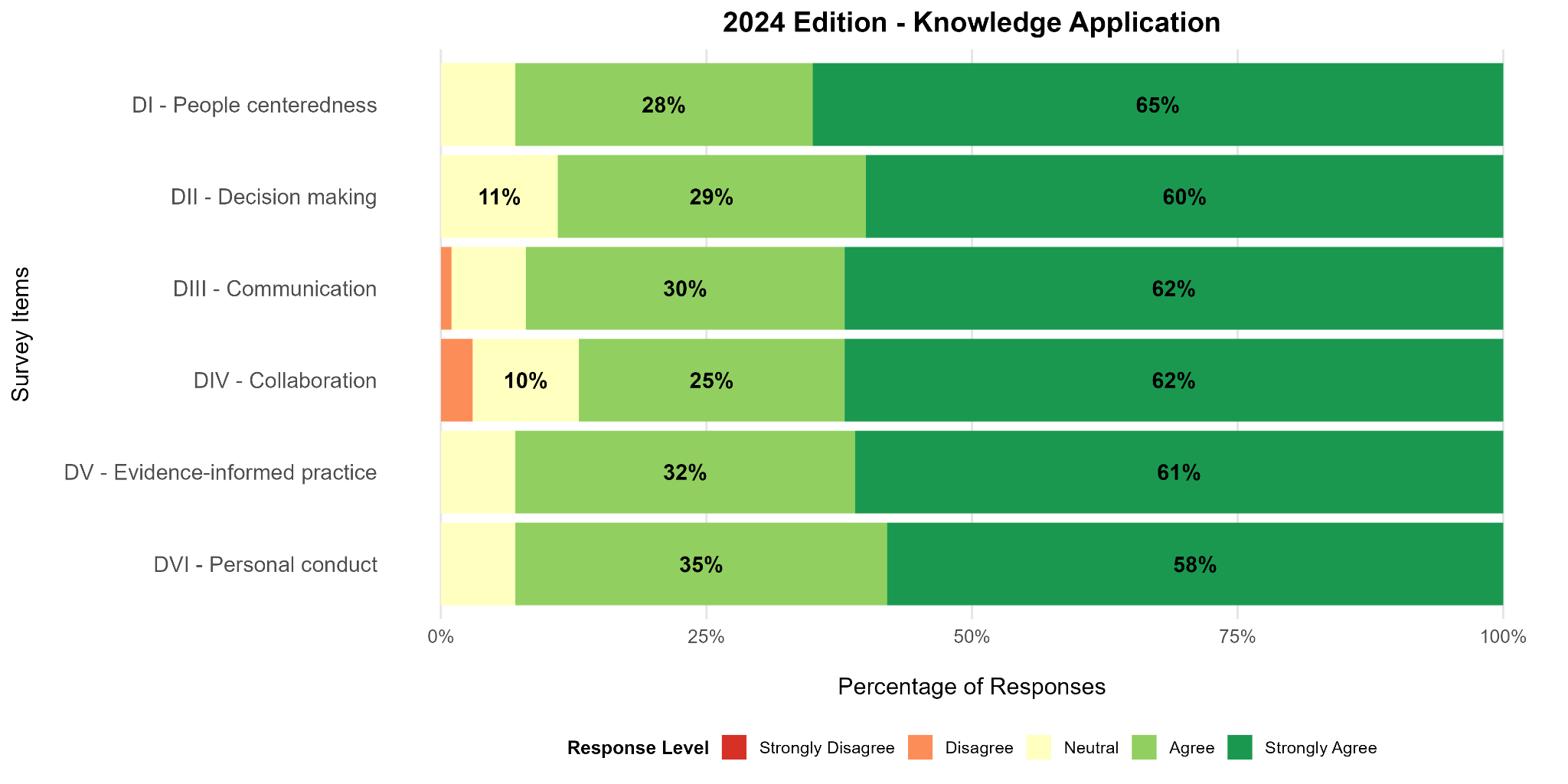


*For readability purposes, options with less than 10% of the answers were omitted.*

***Supplementary Figure 3.*** *Responses of participants of the 2025 edition regarding statements on “Outcomes that Matter” (Domain I).*


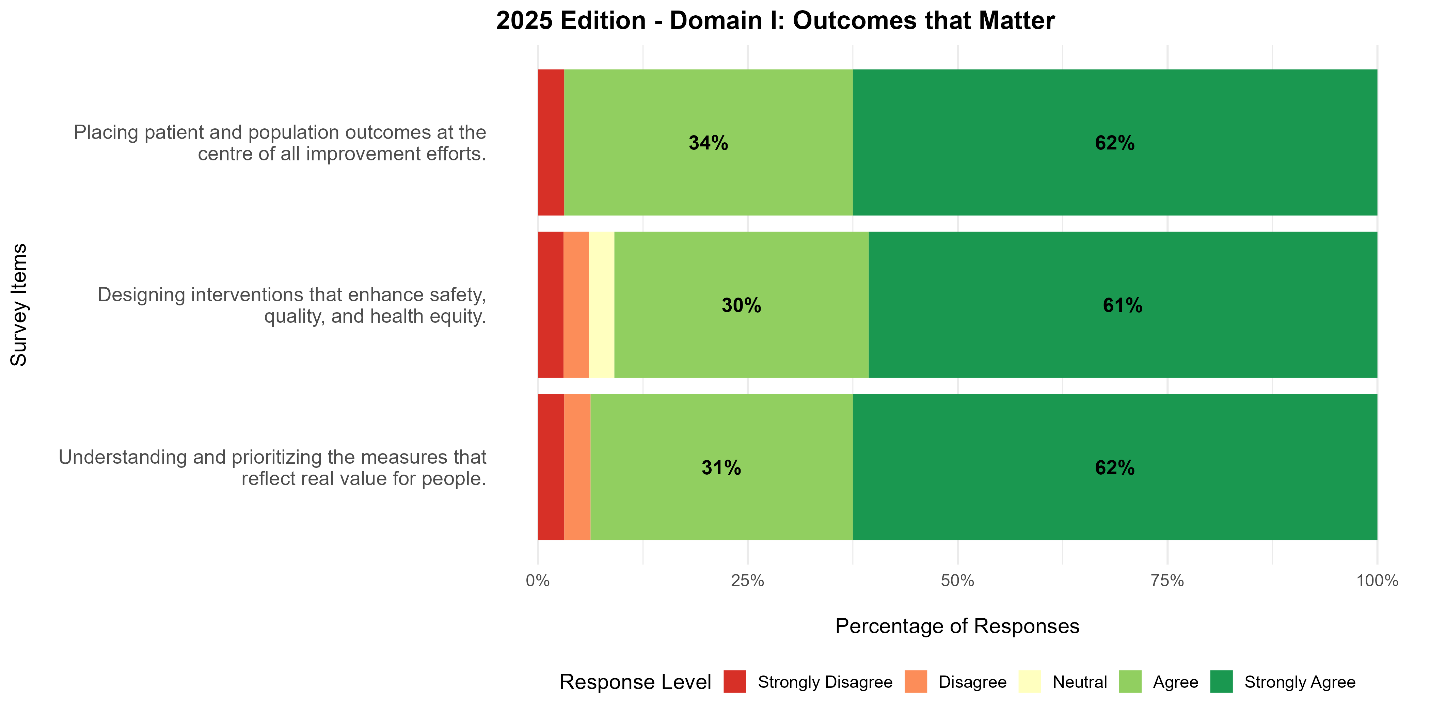


*For readability purposes, options with less than 10% of the answers were omitted.*

***Supplementary Figure 4.*** *Responses of participants of the 2025 edition regarding statements on “Systems Perspective” (Domain II).*


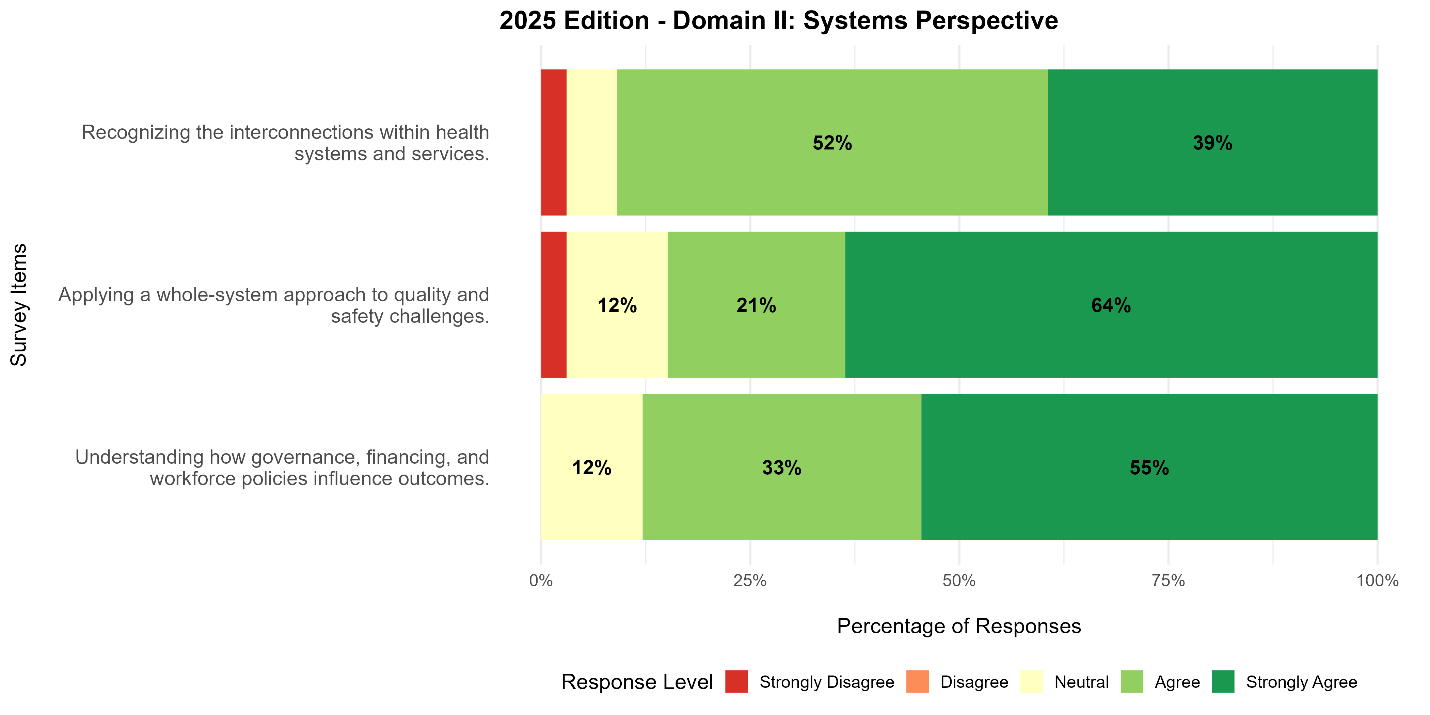


*For readability purposes, options with less than 10% of the answers were omitted.*

***Supplementary Figure 5.*** *Responses of participants of the 2025 edition regarding statements on “People, Leadership, and a Drive for Change” (Domain III).*


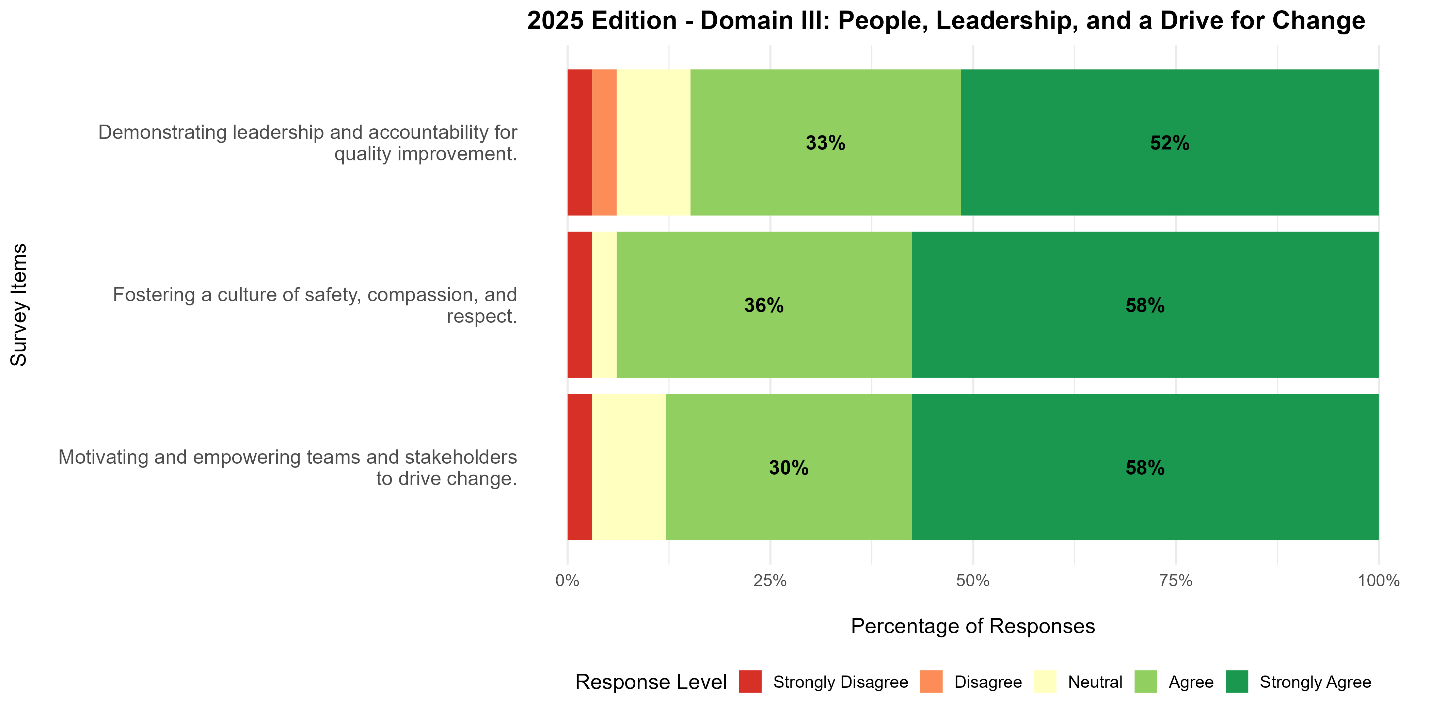


*For readability purposes, options with less than 10% of the answers were omitted.*

***Supplementary Figure 6.*** *Responses of participants of the 2025 edition regarding statements on “Data and Transparency” (Domain IV).*


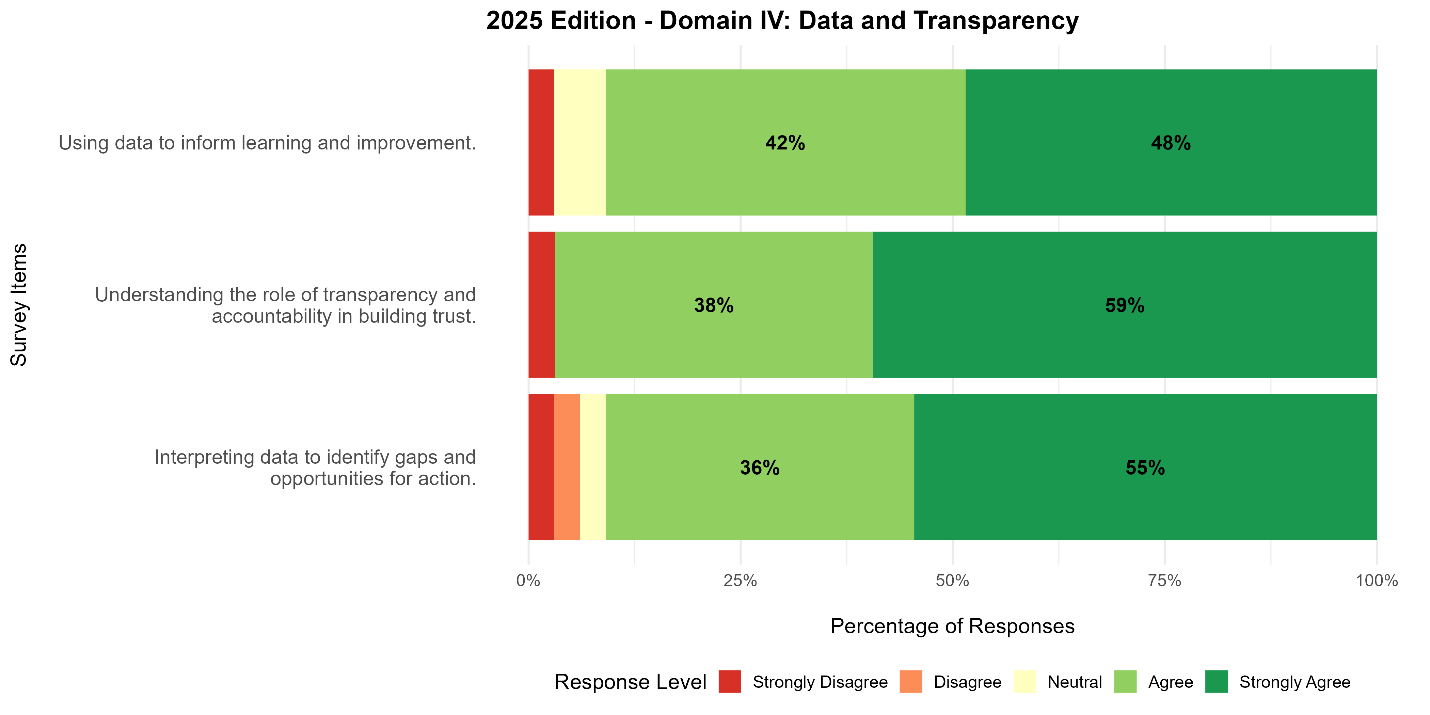


*For readability purposes, options with less than 10% of the answers were omitted.*

***Supplementary Figure 7.*** *Responses of participants of the 2025 edition regarding statements on “Digital Solutions and Innovation” (Domain V).*


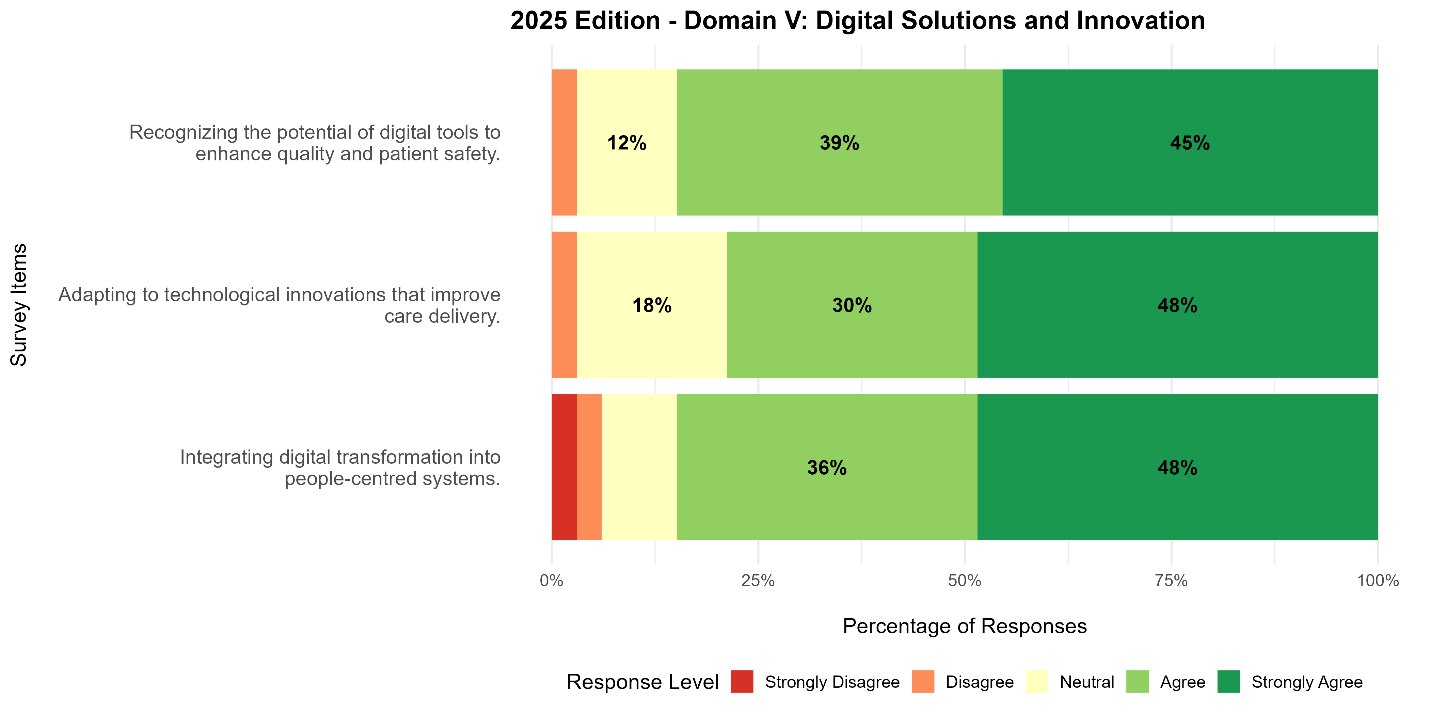


*For readability purposes, options with less than 10% of the answers were omitted.*

***Supplementary Figure 8.*** *Responses of participants of the 2025 edition regarding statements on “Collaboration” (Domain VI).*


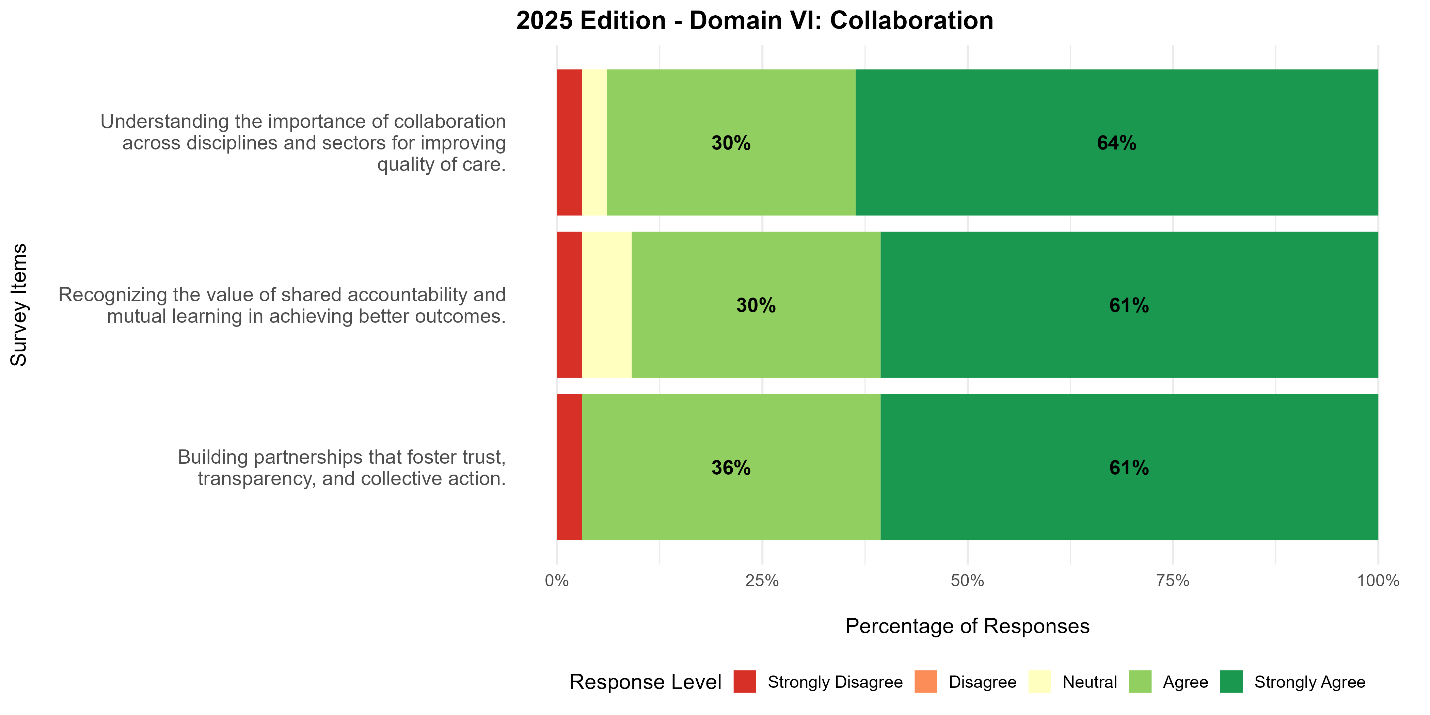


*For readability purposes, options with less than 10% of the answers were omitted.*
